# The enhancement of microbial fuel cell performance by anodic bacterial community adaptation and cathodic mixed nickel–copper oxides on a graphene electrocatalyst

**DOI:** 10.1186/s43141-021-00292-2

**Published:** 2022-01-24

**Authors:** Dena Z. Khater, R. S. Amin, M. O. Zhran, Zeinab K. Abd El-Aziz, Mohamed Mahmoud, Helmy M. Hassan, K. M. El-Khatib

**Affiliations:** 1 Chemical Engineering & Pilot Plant Department, Engineering and Renewable Energy Research Institute, National Research Centre, 33 El-Buhouth St., Dokki, Cairo, 12311 Egypt; 2 Faculty of Science (Girls), Botany and Microbiology Department, Al-Azhar University, Nasr City, Cairo, Egypt; 3 Water Pollution Research Department, National Research Centre, 33 El-Buhouth St., Dokki, Cairo, 12311 Egypt; 4 Microbial Chemistry Department, National Research Centre, 33 El-Buhouth St., Dokki, Cairo, 12311 Egypt

**Keywords:** Microbial fuel cell, NiO–CuO/G, Linear Sweep Voltammetry, Electrocatalyst, Activated sludge

## Abstract

**Background:**

Although microbial fuel cells (MFCs) represent a promising technology for capturing renewable energy from wastewater, their scaling-up is significantly limited by a slow-rate cathodic oxygen reduction reaction (ORR) and the development of a resilient anodic microbial community. In this study, mixed transition metal oxides of nickel and copper (Ni and Cu), supported on a graphene (G) (NiO–CuO/G) electrocatalyst, were synthesized and tested as a cost-effective cathode for ORR in MFCs. Electrochemical measurements of electrocatalyst were conducted using a rotating disk electrode (RDE) and linear sweep voltammetry (LSV) in a neutral electrolyte, and compared with a benchmark Pt/C catalyst. Furthermore, the long-term performance of the as-synthesized electrocatalyst was evaluated in a single-chamber MFC by measuring organic matter removal and polarization behavior. The successful enrichment of electroactive biofilm was also monitored using transmission electron microscopy and the Vitek2 compact system technique.

**Results:**

When compared with the benchmark platinum cathode, the NiO–CuO/G electrocatalyst exhibited high selectivity toward ORR. The rotating disk electrode (RDE) experiments reveal that ORR proceeds via a 4-electron ORR mechanism. Furthermore, the NiO–CuO/G electrocatalyst also exhibited a high power density of 21.25 mW m^−2 ^in an air-cathode MFC, which was slightly lower than that of Pt/C-based MFC (i.e., 50.4 mW m^−2^). Biochemical characterization of the most abundant bacteria on anodic biofilms identified four genera (i.e., *Escherichia coli*, *Shewanella putrefaciens*, *Bacillus cereus*, and *Bacillus Thuringiensis/mycoides*) that belonged to *Gammaproteobacteria*, and *Firmicutes*phyla.

**Conclusions:**

This study demonstrates that the NiO–CuO/G cathode had an enhanced electrocatalytic activity toward ORR in a pH-neutral solution. This novel mixed transition metal oxide electrocatalyst could replace expensive Pt-based catalysts for MFC applications.

**Supplementary Information:**

The online version contains supplementary material available at 10.1186/s43141-021-00292-2.

## Background

Microbial fuel cells (MFCs) are anaerobic biotechnology systems that convert the stored chemical energy in waste streams into electricity through the metabolic pathway of microbial communities [1, 2]. The bioelectrochemical reactions that generate electricity in MFCs involve the oxidation of organic matter in the anode chamber and the reduction of electron acceptors (ideally oxygen) in the cathode chamber. The hallmark of an MFC is the ability of electroactive biofilms (known as electricigens or anode-respiring bacteria) to oxidize organic matter and respire resultant electrons to anode surfaces, which are then transferred to the cathode via an external circuit. This sticky biofilm matrix consists of extracellular proteins, sugars, and a complex bacterial milieu [3]. Concurrently, generated protons (H^+^) move into the electrolyte (leading to a negative anode potential) via a semi-permeable ion exchange membrane, where the reduction of an electron acceptor (ideally Oxygen) occurs to form H_2_O [4, 5].

The major limiting issues affecting MFCs efficiency is the low power output, which currently restricted due to either the cathodic reaction efficiency or high internal resistance [6]. Therefore, it is essential to categorize the foremost limiting parameters that affecting its performance. These are can be classified into three categories: (1) system architecture, (2) biological conditions, and (3) operational conditions. These parameters include anodic microbial communities, mediators, substrate types, catalyst materials, and membranes. Standard operating conditions are facilitated by pH, external resistance, temperature, ionic concentration, and the catholyte aeration flow rate. Thus, because of these limiting parameters, the technological implementation of MFCs for industrial, and social applications is restricted [7, 8].

However, considerable research has sought to address these limitations [7]. A commonly used low-cost design is the air-cathode, single-chamber MFC, which lacks an ion exchange membrane between anode and cathode. When compared with other MFC configurations, the air-cathode MFC approach is a promising option for wastewater treatment owing to the availability and high redox potential of electron acceptors (i.e., air) [9]. For instance, Ieropoulos et al. [10] demonstrated that mediator-less, single-chambered MFCs (SCMFCs) were advantageous for wastewater treatment and power generation when compared with two-chambered systems. This was due to mediator-less SCMFCs’ higher power densities, lower internal resistance, and simple configuration.

Although early studies showed the possibility of using pure cultures in MFCs for generating electricity from simple donor substrates, mixed-culture microbial communities offer an ecological advantage for producing electricity when complex waste streams (e.g., real wastewater) are used as the sole substrates [11]. In this context, Watson and Logan [12] compared power production by *Shewanella oneidensis *MR-1 to mixed cultures in a cube-shaped MFC and observed that power production (148 mW m^−2^) was lower than that by mixed cultures (858 mW m^−2^). They reported that mixed cultures were more desirable than pure cultures because of their easy access, broader adaptability of feedstock, simple operating requirements, advantages in practical applications, and high functional redundancy [12]. In terms of pH, Gil et al. [13] detected a pH difference of 4.1 units after a 5-h operation when an initial pH of 7 was used without buffering. The addition of a phosphate buffer (pH 7.0) shifted the MFC pH to less than 0.5 units, and the current output increased by approximately 2-fold. It is likely that the buffer compensated for the slow proton transport rate and improved proton availability for cathodic reactions.

The cathodic catalyst material considered as one of the main limiting issues that plays a noteworthy function in improving the MFCs efficiency due to its direct effect on the oxygen reduction reactions (ORR) [14] since the kinetic rate of ORR in the neutral pH aqueous electrolyte requires high activation energy of approximately 498 kJ mol^−1^ to split the oxygen (O_2_) [15]. Thus, to enhance the efficiency of MFCs, modification of electrode materials using highly effective ORR electrocatalysts is warranted [16]. Although platinum (Pt) has been commonly used as an electrocatalyst because of its high electrocatalytic activity toward ORR [17], its practical applications are restricted by high costs, low stability during long-term operations, and availability [18, 19]. Over the past decade, several studies have replaced expensive Pt electrocatalysts using cost-effective, non-precious metal oxide electrocatalysts, such as Pt-free transition metals oxides, e.g., cobalt (Co), iron (Fe), and manganese (Mn)-base electrocatalysts [20]. These oxides have gained increasing attention owing to their durability, high performance, and relatively low cost [21]. The use of transition mixed metal oxides electrocatalysts as cathode in MFCs demonstrates high catalytic enhancement activities, through which one metal oxide accelerates the cleavage of the O=O bond of molecular O_2_. After that, the cleavage is followed by the migration of the adsorbed O_2_ atoms into the other metal oxide, where the electro-reduction step takes place [22], e.g., the introduction of MFC enhancing metallic oxides such as MnO_X_ [23], Co–Al_2_O_3_–rGO [24], (SSM/Co_3_O_4_) [25], Ppy/MoO 2[26], and Co_0.5_Zn_0.5_Fe_2_O_4_ [27, 28].

To date, only a small number of studies have investigated the use of non-precious nickel (Ni)-based electrocatalysts. Valipour et al. [5] studied the feasibility of Ni nanoparticles on a reduced graphene oxide (RGO) as a high efficient ORR cathode electrocatalysts in SCMFCs with a maximum power density of 581 ± 19 mW m^−2^, which was slightly lower than Pt/C cathode. Moreover, Huang et al. [29] suggested that Ni oxide supported on a carbon nanotube (NiO/CNT) is a promising non-precious metal composite for ORRs in SCMFCs. The system produced a maximum power density of 670 mW m^−2^. An SCMFC with a Pt-Ni nanoparticle alloy dispersed on carboxyl multi-wall CNTs produced a maximum power density of 1220 mW m^−2^ [16]. In addition, Ghasemi et al [28] generated a power density of approximately 94.4 mW m^−2^ with Ni nanoparticles using two cube-shaped chambers. Similarly, Liu and Vipulanandan [30] used Ni nanoparticles for two-chambered MFC application generated a power density of 0.07 mW m^−2^. In another study, Kartick et al*.* [31] investigated a dual-chamber MFC using a Ni nano fiber hybrid electrocatalyst supported on graphene (G) and reported a power density of 34 mW m^−2^. Furthermore, Champavert et al. [32] modified tetra Sulfonated phthalocyanine electrocatalysts with Ni and used them in a high-performance dual-chamber MFC that generated a maximum power density of 21.6 mW m^−2^. Additionally, Mohamed et al. [14] found that Ni nanoparticles had a maximum power density of 1630.7 mW m^−2^ in tested cobalt (Co)- and Cadmium (Cd)-based reagents. In addition, Chaturvedi et al. [33] accomplished the synthesis of different weight ratios of Co–Ni nanoparticles supported on an alumina-graphene oxide (Al_2_O_3_–GO) matrix to determine ORR activity in MFCs. The approach revealed that the Co–Ni(2:1)/Al_2_O_3_–GO catalyst exhibited an improved ORR rate with a maximum power density of 168 mW m^−2^, when compared with 102 mW m^−2^ for Pt/C.

Support materials are also essential for dispersing electrocatalyst particles, preventing self-aggregation, and improving MFC performance. In this regard, G is typically used as electrocatalyst support because of its large specific surface, high electrical conductivity combined with low internal resistance, and high chemical stability and anti-corrosion properties [34]. In addition, it possesses abundant surface functional groups that provide accessible active sites.

In this study, the feasibility of using NiO–CuO/G as a cathode electrocatalyst in MFCs was explored. Physical and electrochemical characterizations of the nano-electrocatalyst and isolated anodic strains were performed. Optimization of different operating conditions for MFCs was evaluated using the nano-electrocatalyst as a cathode, and the biochemical properties of isolated anodic strains were identified using the Vitek2 compact system method.

## Methods

### Chemicals

Hexahydrates of Ni and copper Cu nitrates (Ni(NO_3_)_2_.6H_2_O and Cu(NO_3_)_2_.6H_2_O) were purchased from Fisher Scientific (USA). Graphene (G) powder (average pore diameter 100 ± 10 Å) and 5wt.% Nafion solutions were purchased from Sigma-Aldrich (MO, USA). Pt on carbon Vulcan (30% Pt/C, E-tek, USA) electrocatalyst was obtained from the Fuel Cell Store (TX, USA). All supplies and chemical materials were of analytical grade purity and were used without further purification. All aqueous solutions were freshly prepared using double distilled water.

### Synthesis of NiO–CuO/G electrocatalyst

NiO–CuO/G composites were prepared using a co-precipitation method [29]. In a typical synthesis, the mixed metal salt precursors of Ni(NO_3_)_2_.6H_2_O and Cu (NO_3_)_2_.6H_2_O were dispersed on high surface area G in double-distilled water. Then, pH was adjusted to approximately 10 using the drop-wise addition of 1.0 M NaOH using Benchtop pH meter (Adwa AD1000, Hungary) followed by vigorous stirring for 3 h to avoid agglomeration and to generate a homogeneous dispersion. The resulting black metal-hydroxide precipitate was filtered and washed several times using double-distilled water and adjusted to pH 7. After this, the final product was generated by drying the precipitate at 80 °C (TiTANOX oven, Italy) for 6 h to remove excess water. The dried precipitate was calcinated for 3 h at 300 °C in a muffle furnace (ShinSaeng Scientific, Korea) to form metal oxides. The corresponding mass ratio in the NiO–CuO/G was 30% of NiO–CuO to 70% of Graphene.

### Electrochemical measurements using a rotating disk electrode

Electrochemical measurements were performed at room temperature (25 ± 1 °C) using an electrochemical workstation (PST006 Voltamaster, USA) and a rotating disk electrode (RDE; CTV 101 speed control unit, USA). The working electrode (with a geometrical surface area of 0.196 cm^2^) was either NiO–CuO/G- or Pt/C-deposited thin film on the surface of the glassy carbon electrode (GCE), while Pt wire and Ag/AgCl (Metrohm, Netherlands) were used as counter and reference electrodes, respectively. Before depositing the catalyst layer on the GCE, the GCE was mechanically polished using 0.05 μm alumina slurry on a soft cloth to generate a mirror-like surface. Finally, the GCE was rinsed in double-distilled water and acetone. Following this, 1.0 mg of electrocatalyst was sputtered and mixed with a drop of isopropanol on the GCE. After the isopropanol was dried, a drop of Nafion solution (5%) was pipetted onto the GCE surface to form a homogeneous thin layer. Finally, a second drop of isopropanol was added, and the electrocatalyst film dried at room temperature overnight.

Linear sweep voltammetry (LSV) studies were conducted in a 50-mM phosphate buffer solution (PBS, pH 7.2) at a scan rate of 10 mV s^−1^ in a potential range of (− 1000 to + 1000 mV vs. Ag/AgCl) (equivalent to − 780 to + 1220 mV vs. standard hydrogen electrode (SHE)) with different rotation speeds (i.e., 0 to 2400 rpm). Before electrochemical measurements were conducted, the electrolyte was sparged with ultra-pure oxygen (O_2_) for 30 min. For comparison, all electrochemical measurements were performed in nitrogen-saturated PBS. A schematic summary is presented in Figure S1.

Kinetic parameters were analyzed based on the Koutechy-Levich (K-L) equation derived from RDE studies to calculate electron transfer numbers (n) involved in the ORR process [35] as follows:

1 $$\frac{\mathbf{1}}{{\mathbf{I}}_{\mathbf{d}}}=\frac{\mathbf{1}}{{\mathbf{I}}_{\mathbf{k}}}+\frac{\mathbf{1}}{\mathbf{0.62nFA}{\mathbf{D}}^{{\mathbf{2}}_{/\mathbf{3}}}{\mathbf{c}\boldsymbol{\upnu}}^{-\mathbf{1}\left/\mathbf{6}\right.}{\boldsymbol{\upomega}}^{\mathbf{1}\left/ \mathbf{2}\right.}}$$

Where *I*_*d*_ is the disk current density; *I*_*k*_ is the electrode potential-dependent kinetic current density; ω is the angular momentum (rads^−1^s^1/2^); *n* is the average number of electrons in the catalytic reaction; *F* is Faraday’s constant (96,485 C mol^−1^); *D* and *C* are the diffusion coefficient of dissolved oxygen (1.9 × 10^−5^ cm^2^ s^−1^) and the concentration of dissolved oxygen in 50 mM PBS (1.117 × 10^−6^ mol mL^−1^), respectively; *v* is the kinetic viscosity of the electrolyte (0.01073 cm^2^ s^−^^1^); and *A* is the geometric area of the disk electrode (0.196 cm^2^) [36].

### MFC configuration

All MFC studies were conducted using single-chamber, air-cathode MFCs (6 cm long, 4.6 cm in diameter, total working volume = 100 mL) as described elsewhere [37] and illustrated in Figure S2. Anodes were three-dimensional carbon felt glued to the top of an externally connected anode port with effective dimensions of 2.5 × 2.5 × 0.6 cm and a projected surface area of 18.50 cm^2^. They were positioned on the other side of the cell (Parallel to the cathode) at a distance of ~ 5 cm from the cathode. Gas diffusion carbon cloth electrodes were used as cathode electrodes (6 × 6 cm each; surface area = 16.63 cm^2^) with a catalyst loading of 0.30 mg/cm^2^. Titanium wire were used as current collectors for both electrodes.

### Cathode electrodes preparation

Cathode electrodes were prepared as described elsewhere [38]. The catalyst was maintained on the water-facing side of a cathode at a mass loading of 0.3 mg cm^−2^. Before coating, catalyst slurry was prepared by mixing NiO–CuO/G composites with a 5% Nafion solution. The mixture was ultra-sonicated at 60 °C for 30 min and uniformly dispersed onto the carbon cloth surface electrode (mesoporous gas diffusion; Fuel Cell Store, TX, USA). To reach the load of the electrode (0.3 mg cm^−2^), multiple catalyst ink layers were deposited on top of each other. Electrodes were dried at room temperature for 24 h before MFC studies. For comparison, 30 wt.% of a Pt catalyst on carbon Vulcan (surface area = ~ 220 m^2 ^g^−1^; E-Tek, USA) was used as a cathode at a catalyst mass loading of 0.3 mg cm^−2^, using the same procedure as previously described.

### MFC operation and analysis

MFCs were inoculated with aerobic activated sludge from a local municipal wastewater treatment plant (Benha, Egypt) and operated under a fed-batch mode for 60 days to allow biofilms to grow on anode surfaces [39]. MFCs were fed with artificial wastewater containing sodium acetate (2.0 g L^−1^) as the sole organic substrate in 50 mM phosphate buffer solution (BPS) (chemical oxygen demand (COD) concentration = 1472 ± 17 mg/L) supplemented with a 12.5 mL mineral solution and a 12.5 mL vitamin solution. The 50 mM PBS solution contained: NaHCO_3_: 2.5 g/L, NH_4_Cl: 0.2 g/L, KH_2_PO_4_: 13.6 g/L, KCl: 0.33 g/L, NaCl: 0.3 g/L, K_2_HPO_4_: 17.4 g/L, CaCl_2_.2H_2_O: 0.15 g/L, MgCl_2_: 3.15 g/L, and a yeast extract: 1 g/L. All MFC studies were conducted in triplicate to calculate average values.

MFCs were operated in a fed-batch mode at room temperature. They were monitored using a data acquisition system (LabJack U6-PRO, USA) connected to a personal computer. An external resistance of 1000 Ώ was used, unless otherwise stated. Current (mA m^−2^) and power densities (mW m^−2^) were calculated according to Ohm’s law as previously described [5, 37]. Polarization and power curves were plotted using a single-cycle technique by recording the pseudo-steady-state voltage across different external resistances, ranging from 175 KΩ to 50 Ω [40]. Internal resistance (*R*_int_) was determined using linear regression corresponding to the Ohmic zone on the linear section of the polarization curve [41].

Influent and effluent COD concentrations were analyzed according to APHA standard methods for water and wastewater examination [42]. Organic concentrations were calculated as COD removal efficiency (COD R%), which was calculated using the following equation:

2 $$\mathrm{COD}\ \mathrm{R}\%=\frac{{\mathrm{COD}}_{\mathrm{initial}}-{\mathrm{COD}}_{\mathrm{final}}}{{\mathrm{COD}}_{\mathrm{initial}}}\ \mathrm{x}\ 100$$

Where COD_initial_ is the COD concentration in the influent (mg COD/L), and COD_final_ is the COD concentration in the final effluent at the end of MFC batch cycles (mg COD/L).

The Coulombic efficiency (*C*_*E*_) was calculated by normalizing the measured current with respect to the theoretical current based on consumed COD as follows:

3 $${\mathrm C}_{\mathrm E}\left(\%\right)=\frac{{\mathrm C}_{\mathrm P}}{{\mathrm C}_{\mathrm T}}\;\mathrm x\;100$$

Where *C*_*T*_ is the theoretical coulombs and was estimated according to the following formula: *C*_*T*_ = (*F* × *N* × *W* × *V*)/*M*), where *F* is Faraday’s constant (96,485 C mol^−1^), *N* is number of moles of electrons (8 mol mol^−1^), *W* is the daily COD load removed (g L^−1^), *M* is the molecular weight of acetate (59 g mol^−1^), and *V* is the medium volume (100 mL) [43]. *C*_*P*_ is the Coulombs equivalent to the actual current produced during one batch cycle.

### Physical characterization of NiO–CuO/G and anodic biofilms

#### X-ray diffraction (XRD) patterns

In order to determine the physical characteristics of NiO–CuO/G (such as lattice composition and distinctive crystallite size), XRD was performed using an XRD–RIGAKU-D/MAX-PC 2500 X-ray diffractometer fitted with Ni-filtered Cu Kα as the radiation source (λ = 0.154056 nm) at a tube current of 40 mA with a 40-kV voltage. The 2*θ* angular regions were detected at a scan rate of 10° min^−1^ from 10° to 80°. XRD data analyses were conducted using the Materials Studio (Accelrys, USA) software suite Reflex module.

#### Scanning electron microscopy(SEM) analysis of electrocatalysts and biofilms

Anodic biofilm growth characterization on bioanode electrode surfaces was visualized at the end of batch studies using SEM (JEOL JAX-840A, Japan). The anode was fixed in 2.5% (w/v) glutaraldehyde for 4 h. Following fixation, samples were washed three times in DI water and dehydrated in ascending ethanol gradient steps (30% to 100% with 10 min for each step) to avoid artifact drying. Finally, samples were sputtered with gold and imaged using SEM at 20 kV. Energy dispersive X-rays (EDX) were mapped using SEM instrumentation.

#### Transmission electron microscopy(TEM) of electrocatalysts and biofilms

The JEOL-JEM 2010 TEM, Japan was used to determine NiO–CuO/G microstructures and particles sizes and to understand the internal morphological characteristics of the isolated bacterial strains. TEM analytical procedures for isolated anodic bacterial strains were conducted under sterile conditions at room temperature (23 °C) by inoculating 100 μL bacterial cultures into a 5-mL sterilized nutrient broth and incubating them at 37 °C for 18–20 h before TEM analysis. Then, samples were fixed in 2.5% glutaraldehyde (w/v) at 4 °C for 10 min. Before TEM imaging, harvested bacterial cells were deposited on the TEM grid, stained with 2% uranyl acetate for 3–5 s on a carbon-coated mesh Cu grid, and air-dried. The Gatan program was used for data processing and particle size measurement [44].

#### Biochemical identification of isolated anodic bacterial communities

The isolated anodic bacterial communities were identified using Vitek 2 manufacturer’s instructions (Biomeriux VITEK-2 Compact Reference Manual-Ref-414532) [45]. A sufficient quantity of anodic biofilm was transferred using a sterile swab into a polystyrene test tube containing 3 mL sterile saline, and mixed in a suspension well. Turbidity was adjusted to the equivalent of 0.5–0.63 McFarl and turbidity units using a turbidity meter (VITEK®2 DensiCHEK™, France). The biofilm suspension was incubated in a vacuum chamber station with data collected at different time intervals to measure suspension turbidity and/or by-products from donor substrate metabolism. Finally, raw data were processed using a special algorithm to eliminate false readings.

## Results

### Physical characterization of NiO–CuO/G

**Fig. 1:**
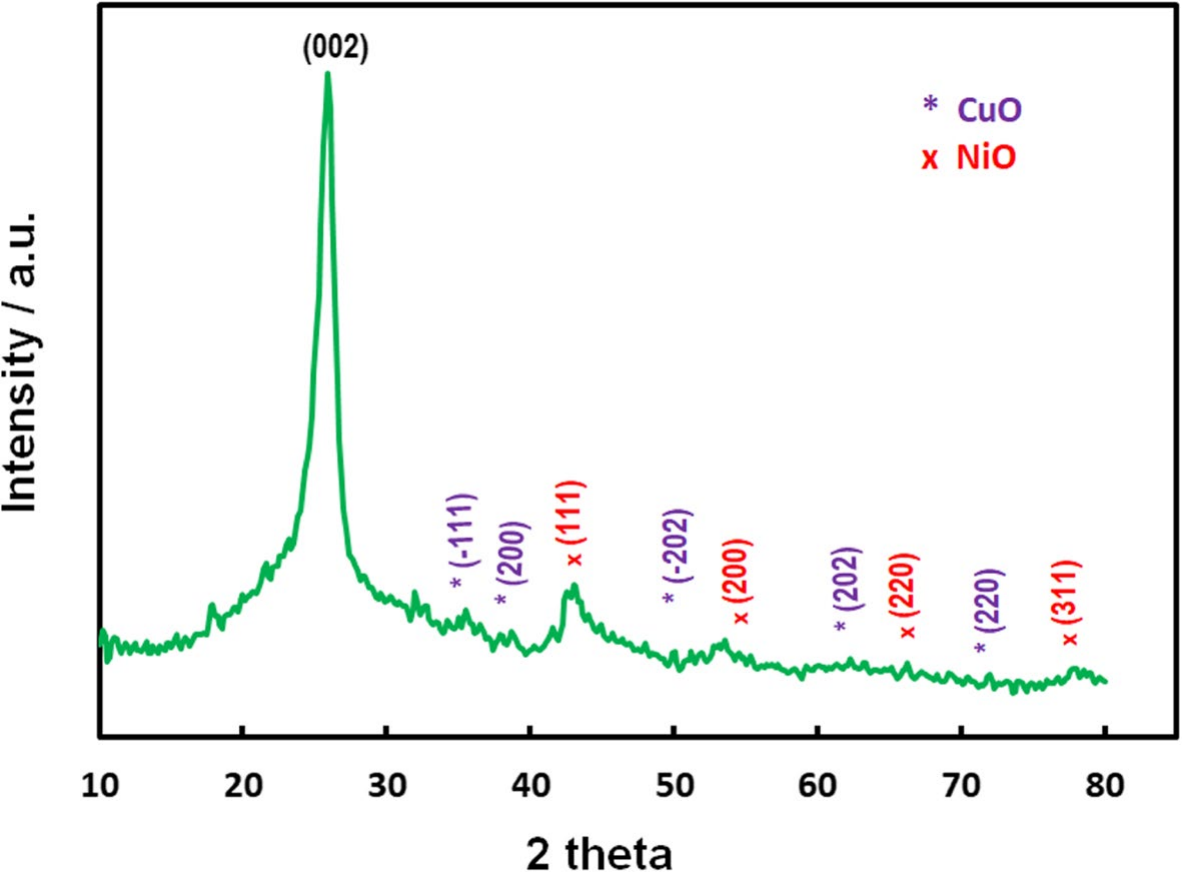
XRD pattern of NiO–CuO/G electrocatalyst

The crystalline features and structural properties of the NiO–CuO/G composite were examined using XRD analysis (Fig. 1). A sharp and powerful diffraction peak was observed at a 2*θ* value of 25.914° (002 plane). Moderately wide distinguished diffraction peaks of four primary planes occurred at 2*θ* values of ~ 43.04°, 54.56°, 64.96°, and 78.88°. In addition, five broad peaks were observed at 2*θ* values of 35.60°, 38.70°, 48.80°, 58.40°, and 74.10°.

**Fig. 2:**
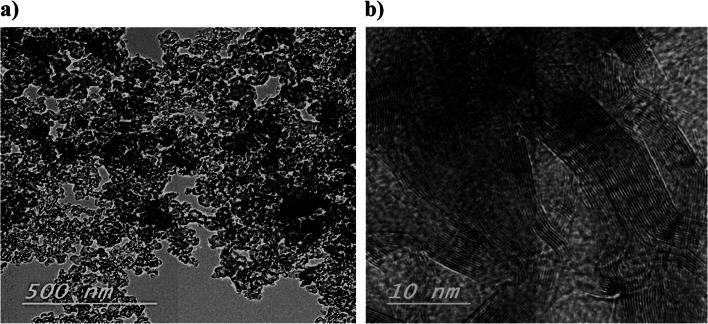
TEM image of the NiO–CuO/G electrocatalyst at **a** magnification of 500 nm and **b** magnification of 10 nm

TEM analysis was performed to characterize NiO–CuO/G composite microstructures and deep surface morphology (Fig. 2). Large quantities of NiO–CuO metal particles had aggregated along with G to form an agglomeration of the electrocatalyst on the G surface (dark spots). High-resolution TEM images of the NiO–CuO/G composite showed hexagonal graphitic edges with parallel alignment to the longitudinal axis of graphitic layers, with typical crumpled structures consisting of several G layers [46].

**Fig. 3:**
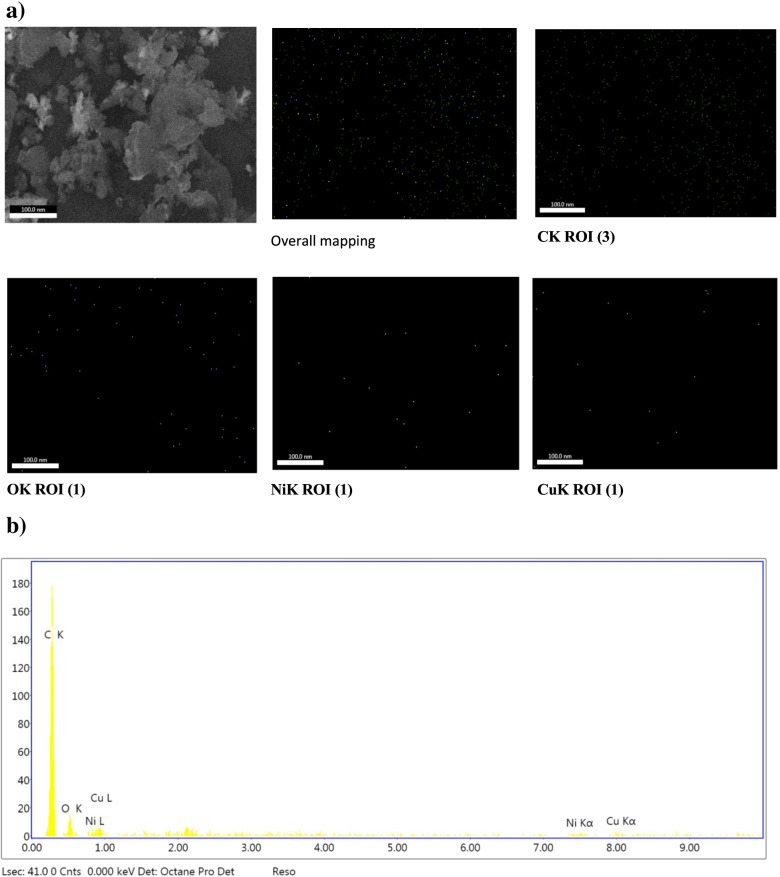
**a**) Energy dispersive X-ray (EDX) mapping analysis and **b**) EDX spectrum of the NiO–CuO/G electrocatalyst

SEM was used to study surface morphology and electrocatalyst composition (Figure S3). The distribution of synthesized composites was observed as granular patterns on the G surface. In addition, a large number of small particulates (white spots) with random aggregation (packed uniformly) was observed on wrinkled and crumpled G structures (gray area). EDX data, in addition to elemental mapping images (Fig. 3), indicated that carbon, oxygen, nickel, and copper were components of the synthesized electrocatalyst. The weights and atomic percentages of different elements are presented in Supplementary Table S1.

### Electrochemical measurements using a rotating disk electrode

**Fig. 4:**
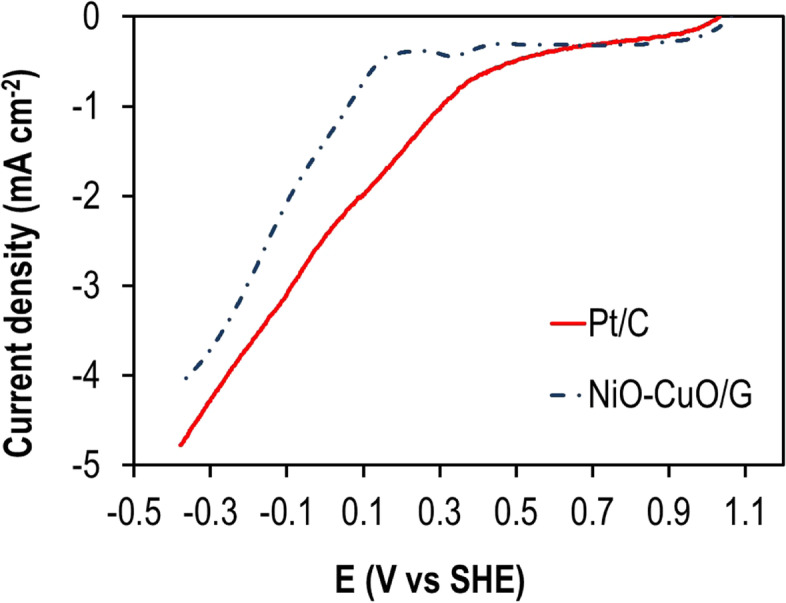
LSV curves of Pt/C and NiO–CuO/G cathodes in O_2_-saturated 50 mM PBS at 25 °C (scan rate = 10 mV s^−1^ and rotation rate = 1200 rpm)

Figure 4 represents the polarization curves of Pt/C and NiO–CuO/G electrodes in O_2_-saturated PBS at a scan rate of 10 mV s^−1^ and a rotation speed of 1200 rpm. The onset potential of Pt/C had a more positive value (400 mV) than that of NiO–CuO/G (163 mV). The current densities at - 0.40 V (vs. SHE) were 4.16 and 4.78 mA cm^−^^2^ for NiO–CuO/G and Pt/C, respectively.

**Fig. 5:**
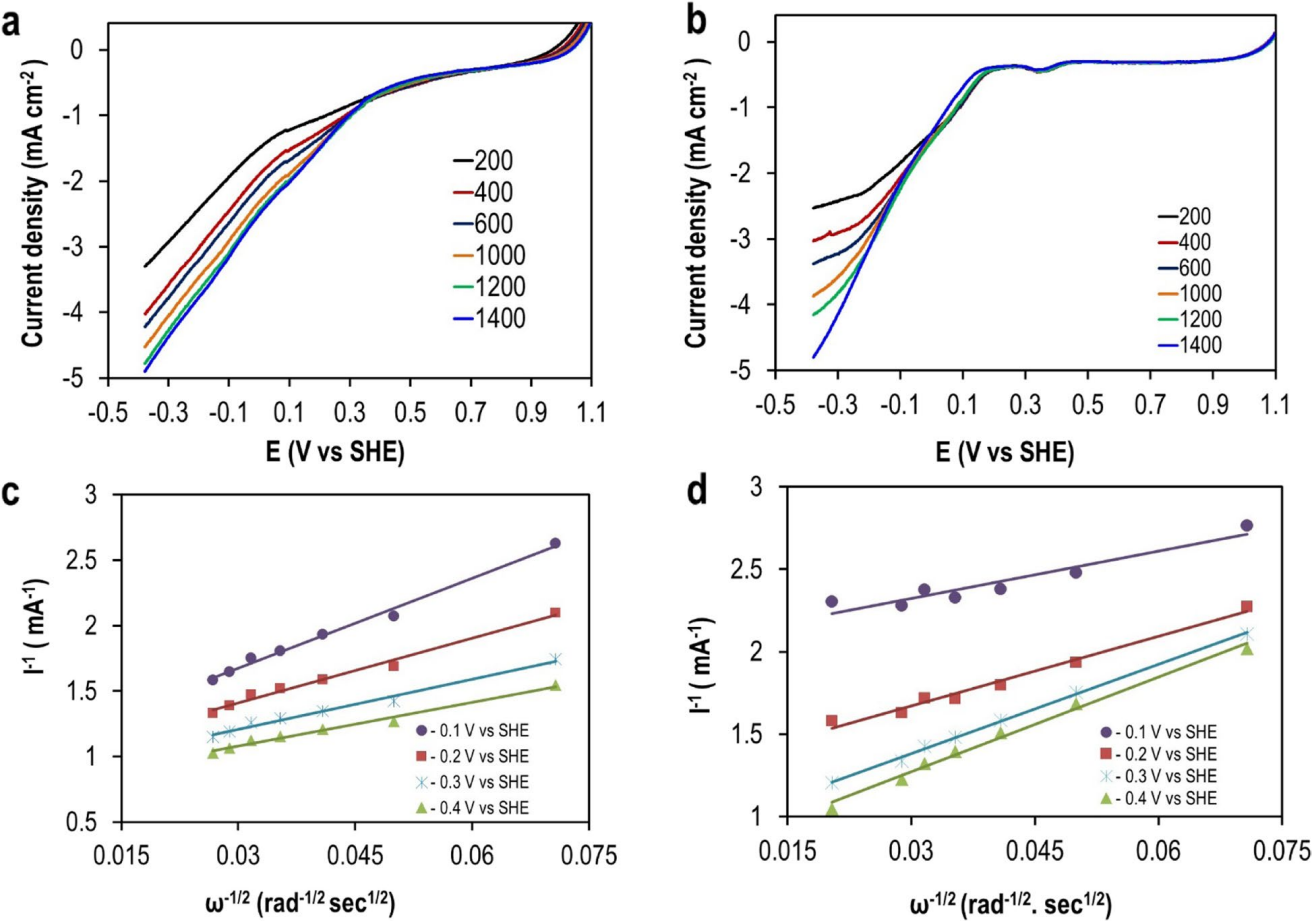
LSV curves obtained from RDE measurements of ORR on **a** Pt/C and **b** NiO–CuO/G in O_2_-saturated 50 mM PBS at various rotation speeds at a scan rate of 10 mV s^−**1**^. Koutecky–Levich (K–L) plots of **c** Pt/C and **d** NiO–CuO/G at different cathodic potentials

To explore the diffusion zone of current densities of Pt/C and NiO–CuO/G, LSV was performed at different rotational speeds, ranging from 200 to 2400 rpm in O_2_-saturated PBS at a scan rate of 10 mV s^1^ (Fig. 5). From Pt/C LSV curves, the reduction current density increased from − 3.22 to − 4.97 mA cm^−2^ with an increase in rotation speed from 200 to 2400 rpm and decreased the potential scan to more negative values. When compared with the benchmark Pt/C electrocatalyst, the NiO–CuO/G electrocatalyst exhibited similar behaviors; the reduction current density increased from − 2.22 to − 4.87 mA cm^−2^ at a potential range of − 0.10 to − 0.40 mV.

RDE polarization curve data were used to plot K–L relationships, where the inverse current density (*j*^*−1*^) was directly related to the inverse square root of the rotation rate (ω^−1/2^) at different potential values (i.e., − 100 to – 400 mV vs. SHE) (Fig. 5c, d)**.** The K–L plots for both electrocatalysts were distinctly parallel and linear; this signified an enhanced electrocatalytic behavior toward ORR (kinetically more facile). The number of electrons (*n*) transferred with ORR was determined from the K–L plot slopes as follows: 3.94 at – 0.10 mV and 4 at – 0.40 mV for Pt/C and ~ 4 at – 0.10 mV and 3.56 at – 0.40 mV for NiO–CuO/G.

### Performance of NiO–CuO/G in MFC

#### Electricity generation

**Fig. 6:**
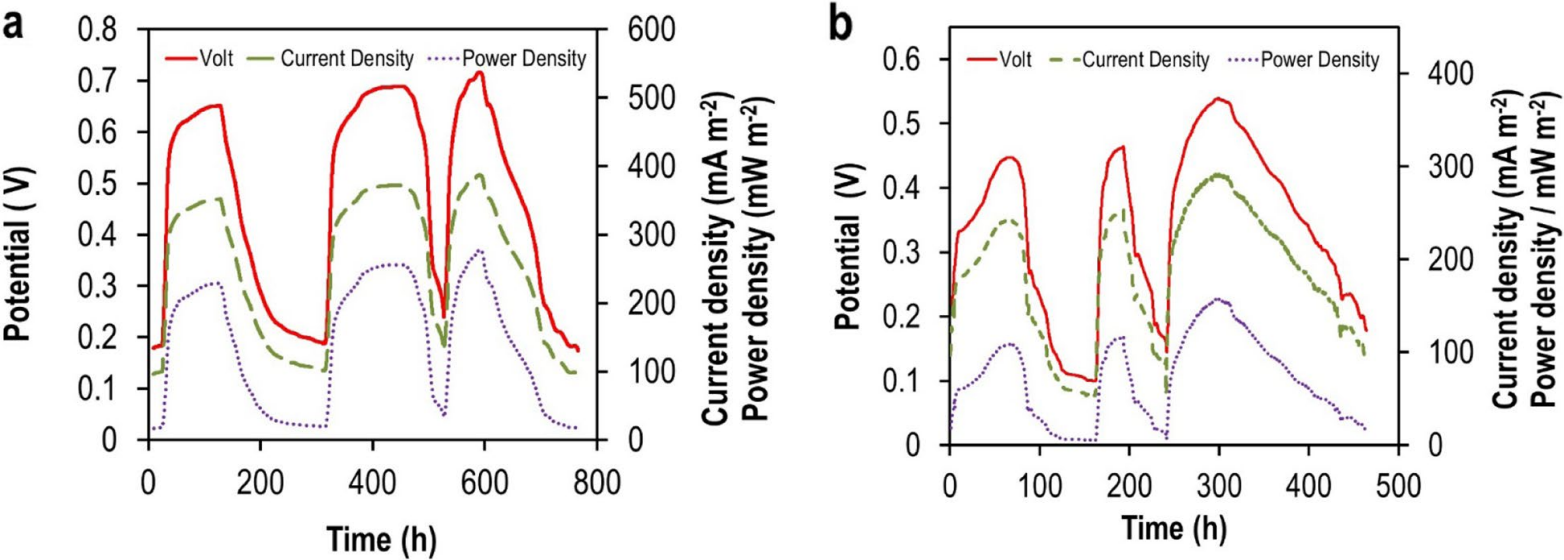
Potential evolution of **a** Pt/C- and **b** NiO-–CuO/G-based MFCs (external resistance = 1000 Ω)

Biochemical and electrochemical reaction rates and the long-term stability of the NiO–CuO/G were evaluated in MFCs under open-circuit voltage conditions (OCV) using acetate as the sole electron donor and compared to a Pt/C-based MFC (Figure S4). Improvements in anodic metabolic activity led to substrate oxidation and an increase in OCV during subsequent cycles. After operating for 62 days, MFCs exhibited a stable OCV of 835 mV for the Pt/C-based MFC and 654 mV for the NiO–CuO/G-based MFC. The closed-circuit cell potential for both MFCs was measured across an external resistance of 1000 Ω. The power density (PD) and potential generation (*V*) curves during three cycles as a function of the current density (*j*) are shown in Fig. 6. The maximum closed-circuit voltage output of the NiO–CuO/G-based MFC was slightly lower than that of the Pt/C-based MFC (541 and 720 mV, respectively).

**Fig. 7:**
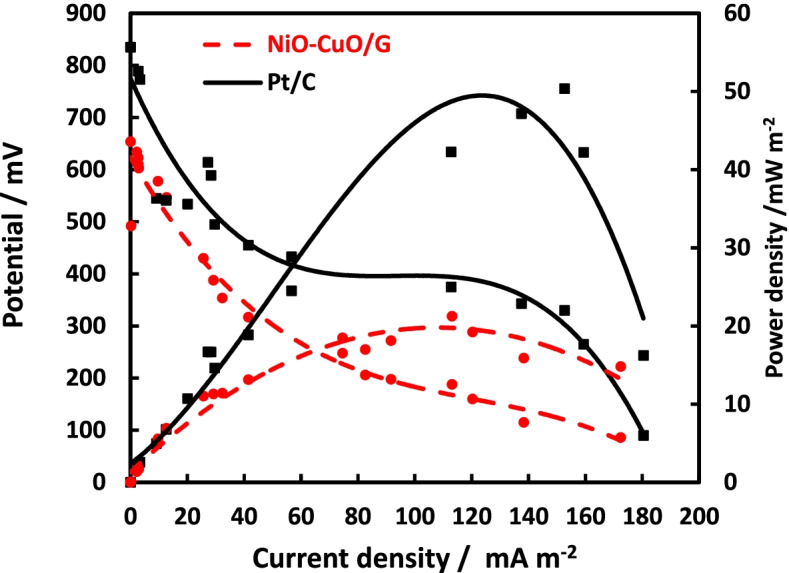
Power density and polarization curves of Pt/C and NiO–CuO/G-based MFCs

The Pt/C cathode exhibited a maximum PD generation value of 50.4 mW m^−2^ at a cell current density of 152 mA m^−2^, which was 2-fold higher than that of the NiO–CuO/G cathode; 21.3 mW m^−2^ at a cell current density of 113.04 mA m^−2^ (Fig. 7). In addition, the NiO–CuO/G had significantly less activation potential loss in the high current region of the range (0–124 mA m^−2^) than that of Pt/C (0–160 mA m^−2^). These results reveal that the major basis for a better catalytic activity of the prepared catalyst is mainly due to improvements in O_2_ mass transfer [47]. The internal resistance of NiO–CuO/G (1120 Ω) was slightly higher than Pt/C (947 Ω).

#### Organic matter removal

For the NiO–CuO/G-based MFC, the COD removal percentage was (92% ± 1.1%), which was slightly lower than that for the Pt/C MFC (93% ± 0.43%). Moreover, the Pt/C displayed significantly higher CE (35% ± 0.69%) than that of NiO–CuO/G (25% ± 0.71%).

#### Visualization of electroactive anodic biofilms

The surface morphologies of fixed, intact anodic biofilms on carbon felt electrodes and bare carbon felt anodes were investigated using SEM. As illustrated in Supplementary Figure S5, the surface morphology of bare carbon felt was very smooth with multiple carbon fibers crossed over one another, forming a mesh-like structure of approximately 20 μm in diameter. However, the formed anodic biofilm covered the surface and internal pores of the carbon felt anode. Furthermore, bacteria appeared as rod-shaped cells, approximately 1.3 μm long.

**Fig. 8:**
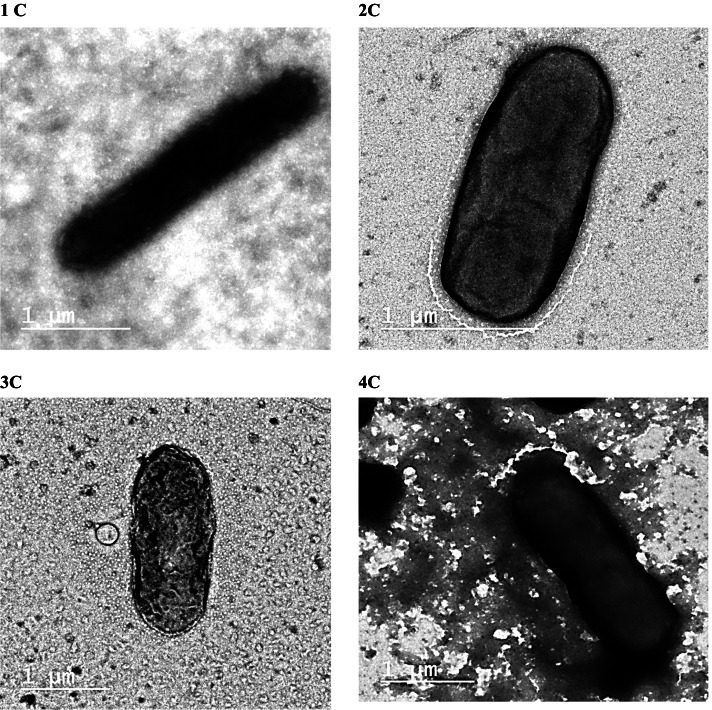
High-magnification transmission electron micrograph image of anodic biofilm

TEM provided information on the internal structures of anodic biofilms, microbial physiology, and the relationship between microbes and minerals. TEM images (Fig. 8) indicate that four isolates (named 1C to 4C) had ultrastructurer rod-shaped surface. Major cellular ultrastructural properties in lipid membranes and the cytoplasm were observed. The cytoplasmic lipid inclusions of isolates were found to be accumulated from the mild amount (1C and 4C) to severe (2C and 3C). Isolates of this bacterial layer had a thickness of approximately 1 μm on their lateral dimension.

#### Biochemical identification of isolated anodic communities

MFC anodic communities were biochemically identified using the Vitek2 compact system method. The analysis revealed the existence of four distinct dominant bacterial classes, with three Gram-negative classes (Table 1), which are commonly found in MFC anodes, e.g., *Gammaproteobacteria* [48] and *Firmicutes* [49]. Interestingly, the high relative abundance of *Gammaproteobacteria* in bioelectrochemical systems is commonly associated with the production of high current and power output [50, 51]. Several members of the *Firmicutes* phylum were found to respire electrons directly onto anode surfaces, such as *Bacillus cereus* and *Bacillus Thuringiensis/mycoides*.

**Table 1:** Biochemical analysis details of anodic biofilm

Characteristic	Isolates			
	2	3	1	4
APPA	−	+	+	+
H2S	−	+	+	+
BGLU	−	−	−	−
ProA	−	+	−	−
SAC	+	+	+	+
ILATk	+	+	−	−
GlyA	−	−	−	−
O129R	+	−	−	−
ADO	−	−	−	−
BNAG	−	+	−	−
dMAL	+	+	+	+
LIP	+	−	−	−
dTAG	+	−	−	−
AGLU	−	−	−	−
ODC	+	+	−	−
GGAA	−	+	−	−
PyrA	−	+	−	−
AGL Tp	−	+	+	+
dMAN	+	−	−	−
PLE	−	−	−	−
dTRE	+	−	−	−
SUCT	+	+	+	+
LDC	+	−	+	+
IML Ta	−	−	+	+
IARL	−	−	(−)	(−)
dGLU	+	−	+	+
dMNE	+	−	(+)	(+)
TyrA	+	+	−	−
CIT	−	−	+	+
NAGA	−	−	(−)	(−)
IHISa	−	−	−	−
ELLM	−	+	+	+
dCEL	−	−	+	+
GGT	−	+	−	−
BXYL	−	−	−	−
URE	−	−	−	−
MNT	−	−	−	−
AGAL	(−)	−	−	−
CMT	+	−	+	+
ILATa	−	−	−	−
BGAL	+	−	+	+
OFF	+	−	−	−
BALap	−	−	−	−
dSOR	**+**	−	−	−
5KG	−	−	**+**	**+**
PHOS	−	+	**+**	**+**
BGUR	−	−	−	−
**Probable identity**	***Escherichia coli***	***Shewanella putrefaciens***	***Bacillus cereus***	***Bacillus Thuringiensis/mycoides***

*+* positive, − negative, *V* variable

## Discussion

In this study, the NiO–CuO/G electrocatalyst was developed as a promising cathode catalyst for MFC applications. Successful catalyst preparation was confirmed using XRD, TEM, SEM, and EDX analyses. First, XRD data revealed that the crystalline nature of the graphite framework supported the electrocatalyst [24]. Furthermore, the C (002) peak became softer as the metal particles on the G fill-up the diffraction toward the composites, leading to a G peak reduction. The 2*θ* values indicated the presence of crystal planes (111), (200), (220), and (311) of NiO and CuO, which were assigned to (− 111), (200), (− 202), (202), and (220) planes. Thus, mixed metal oxides could exist as NiO and CuO, suggesting the effective synthesis of a NiO/CuO composite [52, 53]. The representative peaks (Fig. 1) confirmed the with successful deposition of NiO and CuO on G [54]. This deposition may have been due to G’s elevated surface area and conductivity with available active sites, which may have enhanced the bioelectrochemical efficiency of NiO–ORR [28]. Second, TEM observations agreed with those by Li et al. [55], who prepared a G–Co/Ni composite on carbon cloth electrodes(G–Co/Ni–CC). These researchers reported large amounts of small-sized Co/Ni composites randomly deposited on the surface of crumpled G sheets. Third, from SEM and EDX analyses, NiO and CuO were effectively precipitated on G using the in situ preparation method, with percentages close to the nominal ratio of C, O, Ni, and Cu. These findings indicated that NiO and CuO was successfully deposited on the G surface.

The electrochemical characterization of as-synthesized electrocatalyst when compared with the Pt toward ORR in neutral media was evaluated by LSV. These data suggested that NiO–CuO/G considerably enhanced redox reaction performance and exhibited an electrochemical activity toward ORR comparable with Pt. The current density increased with an increase in rotation speed and a decrease in potential scan to more negative values. This was explained by the transmission of steady streams of the bulk solution to the electrode surface during high rotations, whereas the bulk solution that is far from the electrode surface remains well stirred by the convection and the shortened diffusion distance at subjected speeds [29, 56]. Moreover, kinetic analyses (based on the K-L relationship) revealed that the four-electron pathway directly to water mainly dominates the ORR in Pt/C similar to ORR catalyzed by NiO–CuO/GG according to the following equation:

4 $${\mathrm{O}}_{2}+4{\mathrm{H}}^{+}+4{\mathrm{e}}^{\hbox{-}}\to 2{\mathrm{H}}_{2}\mathrm{O}$$

These results demonstrated an improved electron transfer for the NiO–CuO/G electrode. The distribution of electron transfer numbers confirmed that NiO–CuO/G improved the ORR and improved the performance of the cathode in MFCs in neutral media. These data agreed with a previous study in which silver-tungsten carbide (Ag–WC)/C nano-hybrids showed a comparable ORR efficiency to Pt/C in MFCs [35]. This high ORR activity might have been due to synergistic effects between carbon, WC, and Ag nanoparticles. The NiO–CuO/G contributed to an efficient catalytic ORR activity. Therefore, catalytic activity toward ORR was identified from the combined impact of NiO and CuO loaded onto G. Furthermore, the elevated ORR performance of NiO–CuO was due to the high catalytic surface area of NiO–CuO particles, there by facilitating high deposition rates of the catalyst onto G surfaces and the high porosity of the composites. These results provided evidence on the use of cheaper Pt-free mixed metal oxides as electrocatalysts in MFCs, without significantly impeding performance.

The evaluation of electrocatalyst performance in MFCs showed that maximum OCV values were correlated with an increase in reaction rates, thereby allowing the adsorption and diffusion of higher amounts of O_2_ onto the electrocatalyst surface [57]. The performance of the NiO–CuO/G cathode in MFCs was comparable to Pt/C during the biofilm acclimation period (62 days). NiO–CuO/G-based MFCs had a maximum PD of 21.3 mW m^−2^ and *C*_*E*_ of 25% ± 0.71%, which is somewhat close to Pt/C-based MFCs (e.g., PD = 50.4 mW m^−2^ and *C*_*E*_ = 35 ± 0.69). These data suggested that *C*_*E*_ was determined mainly by cathode variations, but they might have been attributable to the ideal properties of the NiO–CuO/G electrocatalyst. The formation of the biofilm is dependent on the intensive development of the bacterial cells at a high COD removal value. It can be presumed that the higher COD removal is directly correlated to the enhanced substrate utilization and the comparatively higher performance with improved power output [24, 28]. The high surface area of G’s uniform distribution and dispersion of the prepared catalyst led to a higher voltage. This led to a higher current output of the system, with higher power output. Thus, the overall reaction of the NiO–CuO/G, with a high COD removal efficiency, revealed that NiO–CuO/G cathode may be efficiently used as an electrocatalyst for MFC applications.

Surface analyses of anodic biofilms using SEM and TEM indicate that bacterial cells were rod-shaped structures indicative of electroactive microorganisms and thus confirming that the generated electricity was due to electroactive biofilms on the surface of anodic electrodes. In addition, the biochemical characterization of anodic bacterial communities revealed a possible mechanism for the isolation of electrochemically active bacteria (EAB). These analyses indicate that the anode itself is supposed to be a pathway through an anaerobic enrichment of anodic biofilm. The approach considers the fundamental properties of living organisms to absorb respiratory energy via electrons. Bacteria use this energy as an alternative to direct respiration in the absence of an electron acceptor.

The literature survey yielded that non-precious Ni-based electrocatalysts generally exhibited good catalytic activity and stability comparable to Pt/C cathode in MFCs (Table 2).

**Table 2:** A comparative study of the performance of MFC between using different nickel-based electrocatalyst

Cathode catalyst	Anode material	Cathode material	Substrate	MFC configuration	Microorganism	Open circuit potential (mV)	closed circuit voltage (mV)	PD_**max**_ (mW.m^**−2**^)	Percentage to Pt cathode (%)	Ref.
NiO–CuO/G	Carbon felt	Carbon cloth	Sodium acetate	Air cathode	Activated sludge	654	541	21.25	42.16	This study
Nickel nanoparticles on reduced graphene oxide	Graphite brush	Carbon cloth	Sodium acetate	Air cathode	Anaerobic digester sludge	602	136.8	581	26.4	(Valipour et al. 2016) [5]
Naphthalocyanine on carbon black (NPc/C)	Carbon paper	Carbon paper	Wastewater	Double chamber	Anaerobic digester sludge	602	168	29.7	36.53	(Rae et al. 2011) [58]
Nickel oxide and carbon nanotube composite (NiO/CNT)	Carbon felts	Carbon cloth	Glucose	Air cathode	Acclimated sludge from methane-generating pond	772	380	670	N/A^a^	(Huang et al. 2015) [29]
Pt-Ni alloy Nano particles on Carboxyl multi-wall carbon nanotubes (Pt-Ni/MWNT)	Carbon cloth	Carbon cloth	Glucose	Air cathode	Pre-domesticated bacteria from another double chamber MFC	740	570	1.22	86.8	(Yan et al. 2012) [16]
Ni-tetra sulfonated phthalocyanine	Stainless steel foam was modified with rGO	Carbon felt	Sodium acetate	Double chamber	A mixture of compost garden’s leachate			24.8	N/A^a^	(Champavert et al. 2015) [32]
Graphene/nickel hybrids	Graphite plate	Graphite plate	Waste water	Dual chamber	Wastewater			34	N/A^a^	(Kartick et al. 2016) [31]
Nickel nanoparticles	Carbon paper	Carbon paper	Glucose	Dual chamber	Palm oil mill effluent anaerobic sludge	751.8		94.4	78.15	(Ghasemi et al. 2013) [28]

*CNT* carbon nanotube, *MWNT* multi-wall carbon nanotubes

^a^ *N/A* not available

Ni-based electrocatalysts could be used as a cathode catalyst in the MFC as evidenced by the high PD in MFCs. The as-synthesized electrocatalyst was found to be more efficient than those reported by Liu and Vipulanandan [30]. These authors used Ni nanoparticles for a two-chamber MFC application that generated a PD of 0.07 mW m^−2^. In another study, Champavert et al. [32] fabricated a carbon felt cathode modified with poly Ni (II) tetra sulfophthalocyanine for a dual-chamber MFC. This generated a PD of 21.6 mW m^−2^, which was comparable to our results (21.3 mW m^−2^). This difference in PD may have been due to several factors, including operational conditions, MFC design, anode type and surface area, differences in bacterial communities, and differences in supporting material for the electrocatalyst and its projected surface area.

Our research provides new perspectives for effective non-precious mixed metal oxide (NiO–CuO/G) cathode electrocatalysts as replacements for noble and costly Pt/C for practical MFC applications. The enhanced electrocatalytic activity of NiO–CuO/G might have been primarily due to its high surface area and synergistic effects between NiO/CuO and G. These synergistic effects provided NiO/CuO surfaces with high quantities of active sites, thereby confirming electrocatalyst stability, electrical conductivity, and enhanced MFC performance.

## Conclusions

To the best of our knowledge, it was the first time to use this combination of mixed metal oxides (NiO–CuO/G) for application in MFC as a cathode electrocatalyst. The successful preparation of NiO–CuO/G electrocatalyst was confirmed by XRD, TEM, SEM, and EDX analysis. The electrochemical characterization showed high selectivity and electrocatalytic activity of the electrocatalyst towards the ORR that follows the four-electron pathway. The efficiency of NiO–CuO/G in MFCs yielded a maximum PD of 21.3 mW m^−2^ with a *C*_*E*_ of 25 ± 0.71%. These results were slightly lower than Pt/C based MFCs (PD = 50.4 mW m^−2^ and *C*_*E*_ = 35 ± 0.69%). The enhanced electrocatalytic activity of NiO–CuO/G may be mainly due to its high surface area and synergistic effect between NiO/CuO and graphene. These synergistic effects provide NiO/CuO surface with large amounts of active sites, resulting in high stability of the electrocatalyst and enhanced electrical conductivity. This enhanced the performance of MFCs. Both SEM and TEM analysis of anodic biofilm showed the rod-shaped structure of electroactive microorganisms, confirming that the generated electricity was due to the formed electroactive biofilm on the surface of the anodic electrode. In addition, the biochemical characterization of the anodic communities reveals a possible pathway for the isolation of EAB via anaerobic enrichment and was primarily anticipated as a tool for selecting EAB consortia. This research provides new perspectives into discovering effective non-precious mixed metal oxides (NiO–CuO/G) cathode electrocatalyst as a replacement for noble and very costly Pt/C for practical applications of MFCs. The future work will be relying on exploring different combinations of transition metal oxides as ORR electrocatalysts, such as nickel along with cobalt or manganese oxides. The performance of these electrocatalysts will be evaluated using different physical and electrochemical techniques. Moreover, their applications in MFC with different constructions and operating conditions will be performed.

## Supplementary Information

**Additional file 1:** **Figure S1.** Illustrating the synthesis of NiO-CuO/G electrocatalysts by precipitation of metal salts precursors and its techniques for the ORR in neutral PBS and its application in MFCs. **Figure S2.** MFC configuration. **Figure S3.** SEM images of 30 wt % NiO-CuO/G composite: (a) high-magnification image and (b) low-magnification image. **Figure S4.** Open circuit potential for a) Pt/C- and b) NiO-CuO/G-based MFCs. **Figure S5.** SEM images of (a) bare carbon felt anode and (b) carbon felt anode after 90 days operation. **Table S1.** Weight and Atomic percentages of elements forming NiO-CuO/G electrocatalyst.

## Data Availability

All data generated or analyzed during this study are included in this published article and its supplementary information files.

## Abbreviations

CE: Coulombic efficiency
COD: Chemical oxygen demand
DI: De-ionized
EDX: Energy dispersive X-ray spectroscopy
GCE: Glassy carbon electrode
LSV: Linear sweep voltammetry
MEC: Microbial electrolysis cell
MFC: Microbial fuel cell
OCV: Open-circuit voltage
ORR: Oxygen reduction reactions
PBS: Phosphate buffer solution
PD: Power density
RDE: Rotating disk electrode
SEM: Scanning electron microscopy
TEM: Transmission electron microscopy
XRD: X-ray diffraction
