# Supplementary material for: The enhancement of microbial fuel cell performance by anodic bacterial community adaptation and cathodic mixed nickel–copper oxides on a graphene electrocatalyst
Source: J Genet Eng Biotechnol. 2022 Jan 24;20:12. doi: 10.1186/s43141-021-00292-2 (PMC8787007; doi:10.1186/s43141-021-00292-2)
Supplement: Supplementary file 1 — Additional file 1: Figure S1. Illustrating the synthesis of NiO-CuO/G electrocatalysts by precipitation of metal salts precursors and its techniques for the ORR in neutral PBS and its application in MFCs. Figure S2. MFC configuration. Figure S3. SEM images of 30 wt % NiO-CuO/G composite: (a) high-magnification image and (b) low-magnification image. Figure S4. Open circuit potential for a) Pt/C- and b) NiO-CuO/G-based MFCs. Figure S5. SEM images of (a) bare carbon felt anode and (b) carbon felt anode after 90 days operation. Table S1. Weight and Atomic percentages of elements forming NiO-CuO/G electrocatalyst. [file 43141_2021_292_MOESM1_ESM.docx]

**Enhancement of microbial fuel cell performance by anodic communities adaptation and cathodic mixed nickel-copper oxides (NiO-CuO) supported on graphene electrocatalyst**

**Supporting Information**

**Number of Figures: 5**

**Number of Tables: 1**
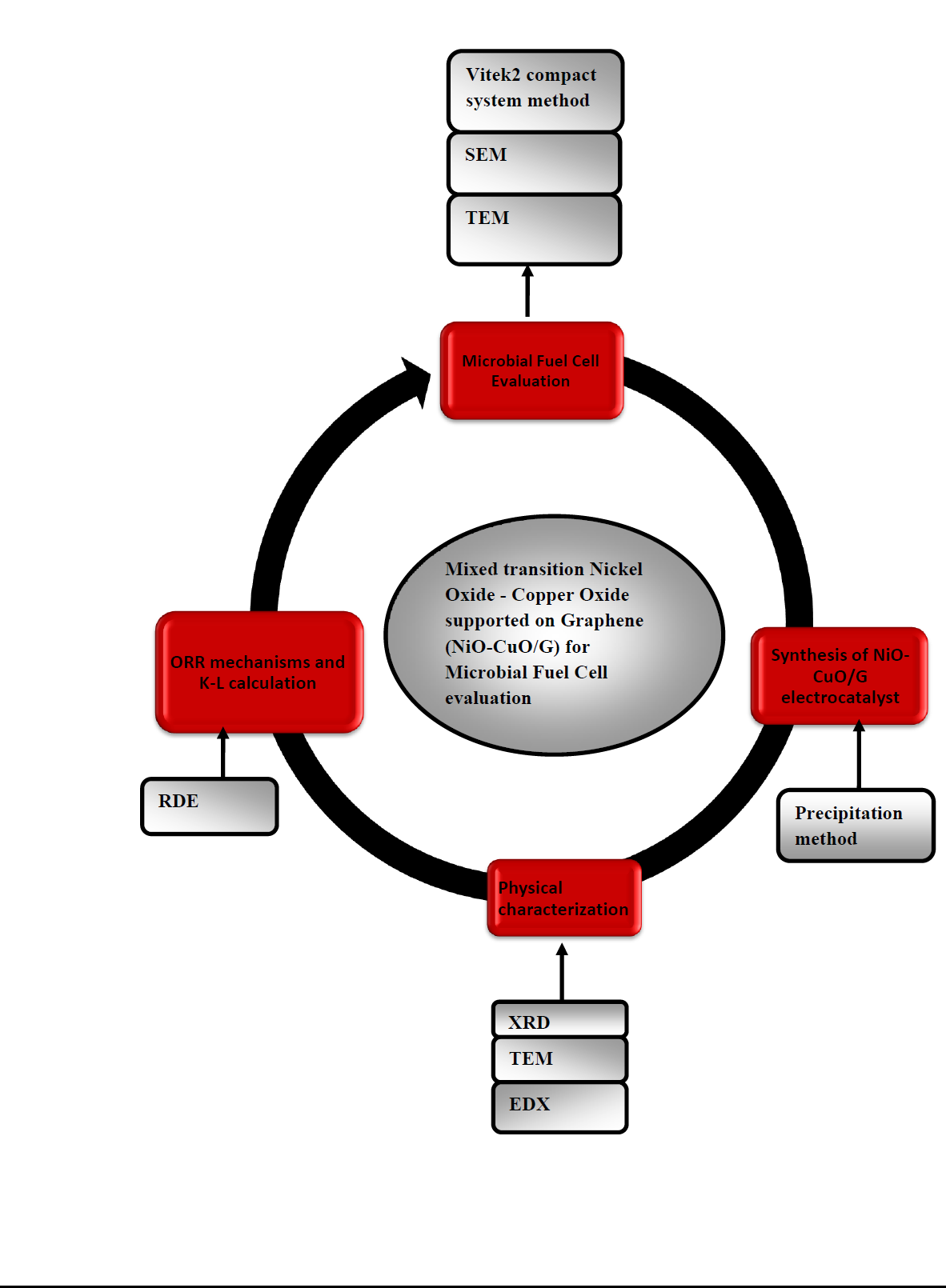


Fig. S1. Illustrating the synthesis of NiO-CuO/G electrocatalysts by precipitation of metal salts precursors and its techniques for the ORR in neutral PBS and its application in MFCs.


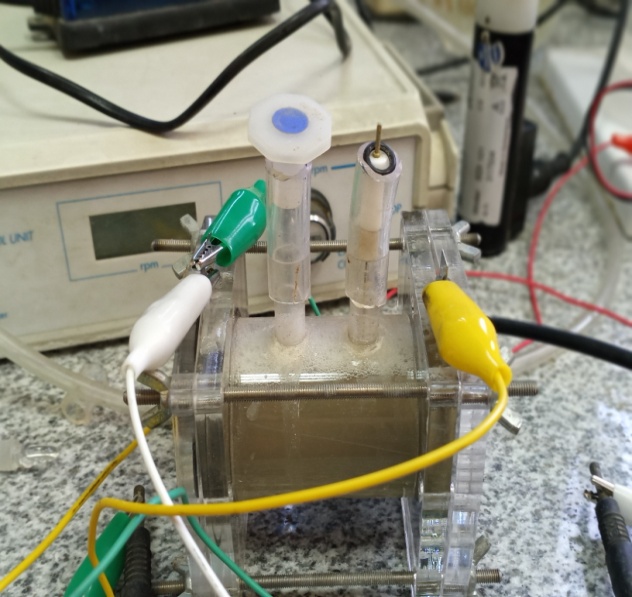


Fig. S2. MFC configuration.

| **a)** | **b)** |  | |
| --- | --- | --- | --- |
| 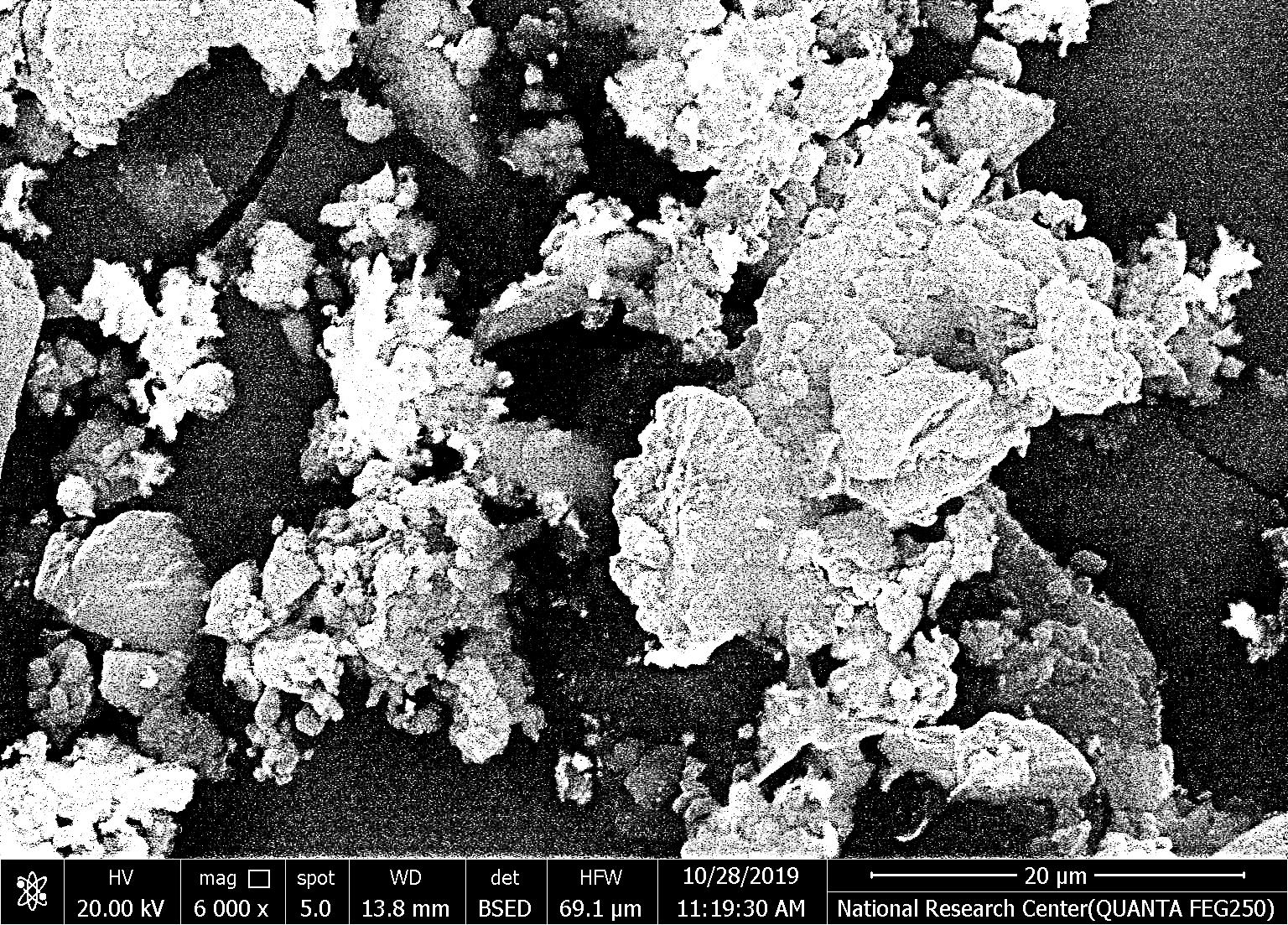 | 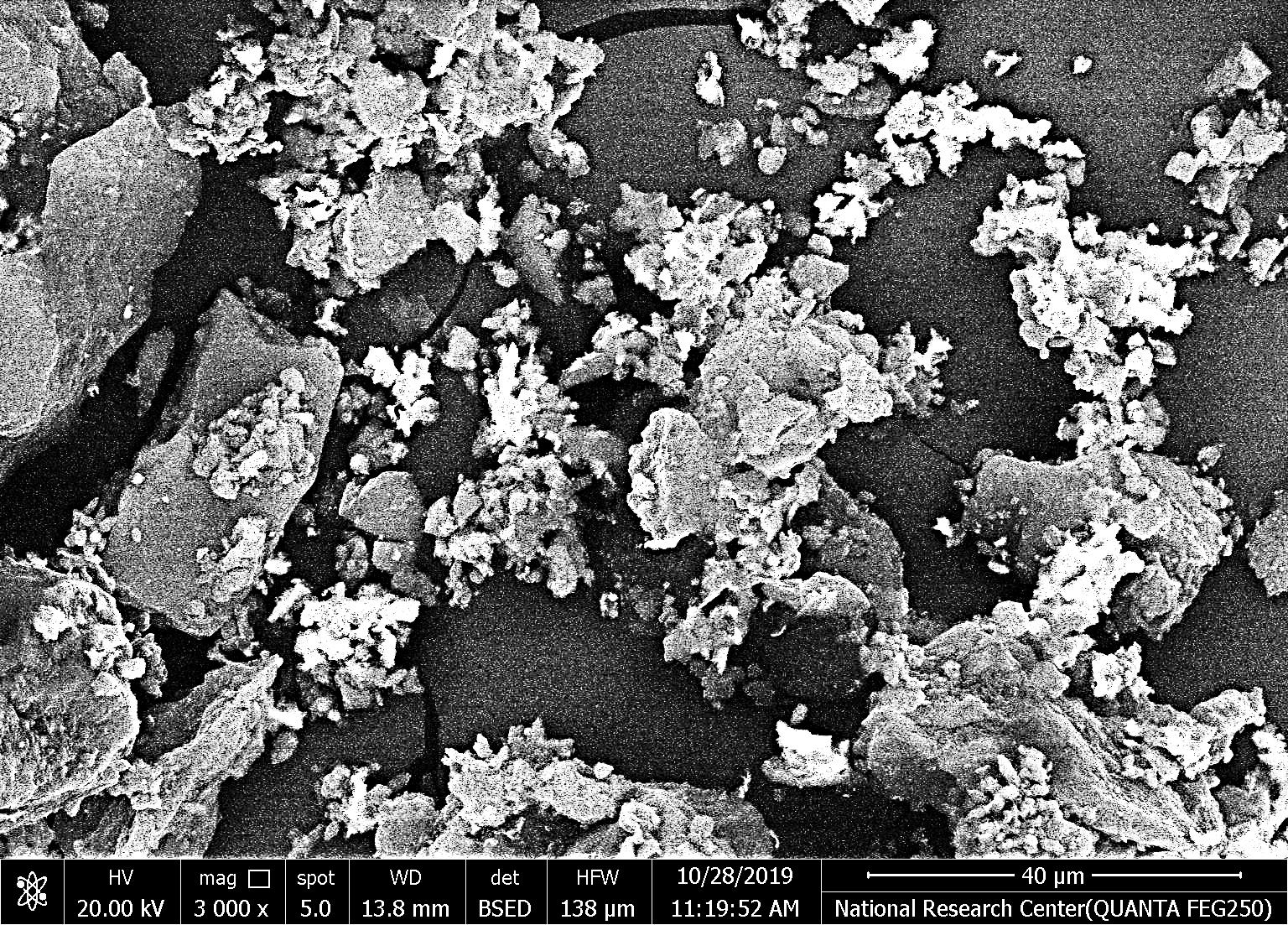 |  |  |

Fig. S3. SEM images of 30 wt % NiO-CuO/G composite: (a) high-magnification image and (b) low-magnification image.

| **a)** | **b)** |
| --- | --- |
| 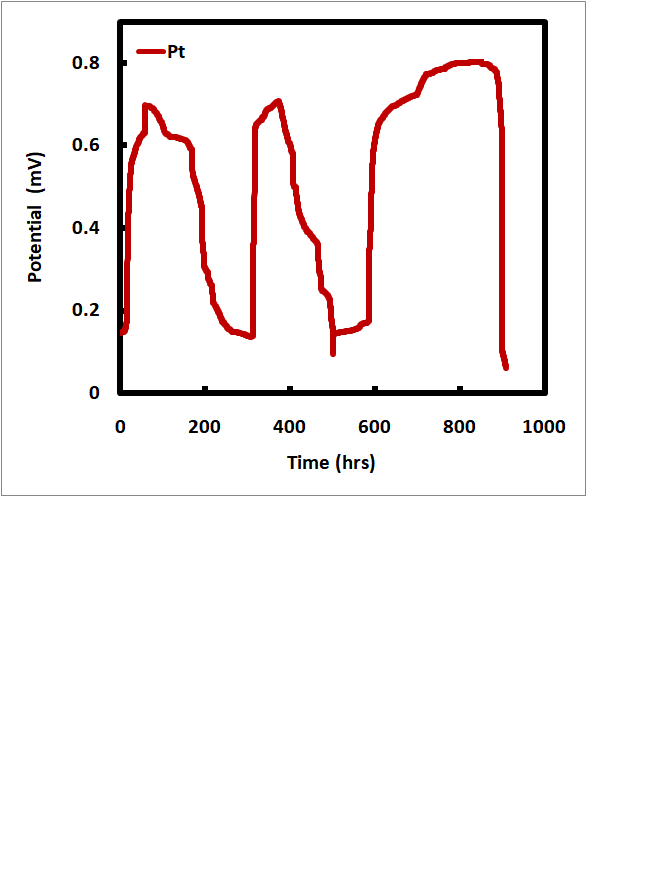 | 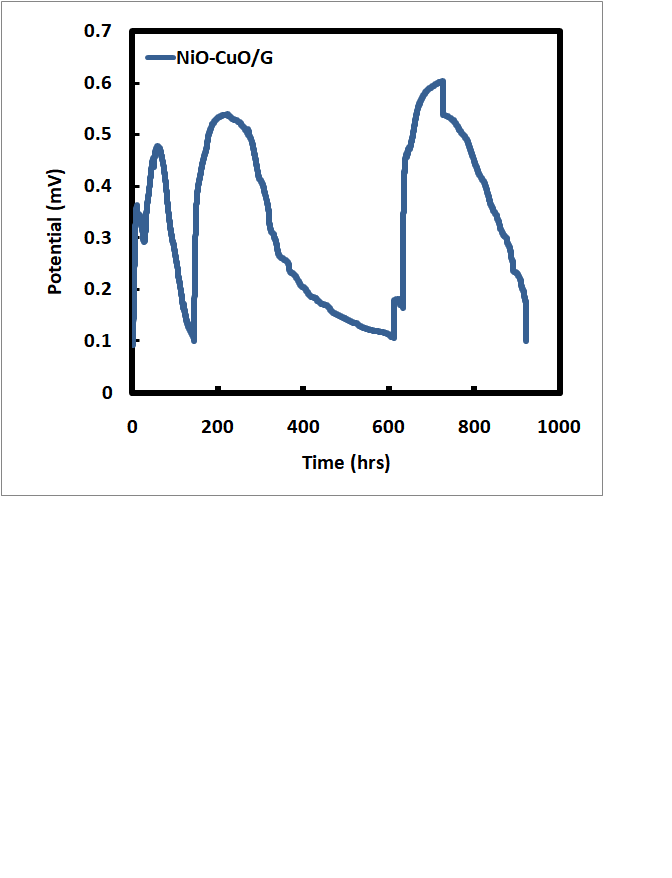 |

Fig. S4. Open circuit potential for a) Pt/C- and b) NiO-CuO/G-based MFCs

| **a)** | **b)**  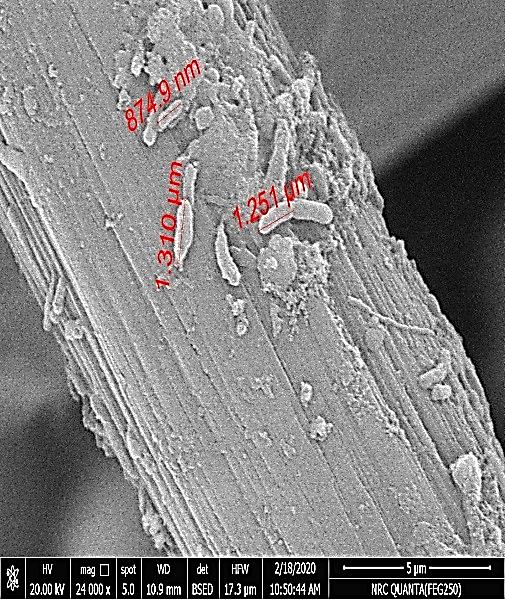 |
| --- | --- |
| 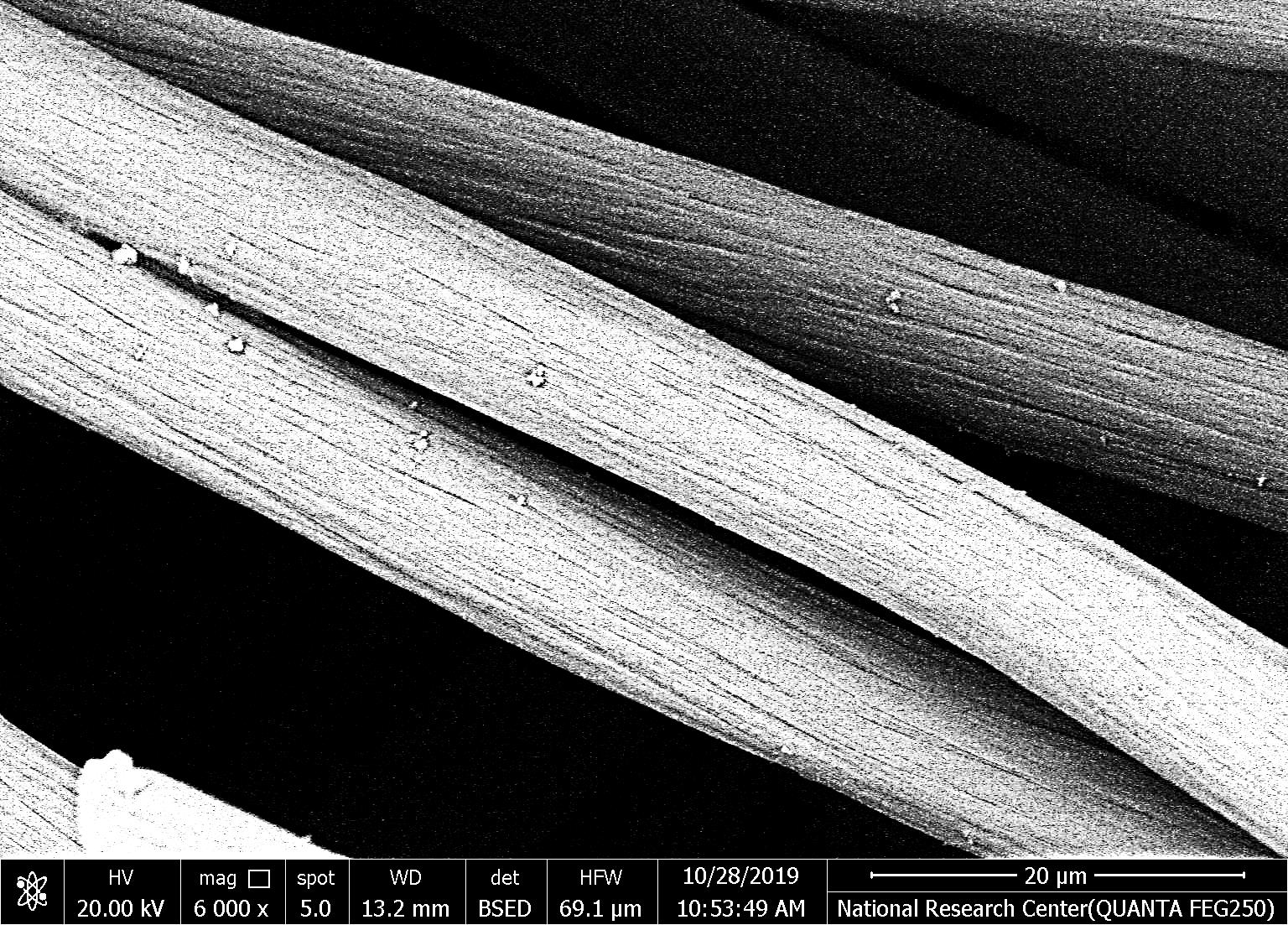 |  |

**Fig. S5** SEM images of (a) bare carbon felt anode and (b) carbon felt anode after 90 days operation.

Table S1. Weight and Atomic percentages of elements forming NiO-CuO/G electrocatalyst

| Element | Weight% | Atomic% |
| --- | --- | --- |
| C K | 77.51 | 84.18 |
| O K | 18.31 | 14.93 |
| NiK | 2 | 0.44 |
| CuK | 2.17 | 0.45 |
